# Clinical, biochemical, and molecular characteristics of Sanfilippo a syndrome (MPS IIIA) in a cohort of Egyptian patients

**DOI:** 10.1186/s13023-025-03971-2

**Published:** 2025-08-25

**Authors:** Ekram Fateen, Soha S. Nosier, Nahla N. Abdel Aziz, Amira M. Radwan, Eman E.A. Mohammed

**Affiliations:** 1 Biochemical Genetics Department, Human Genetics and Genome Research Institute, National Research Centre, Cairo, Egypt; 2 Medical Biochemistry Department, Faculty of Medicine, Damietta University, Damietta, Egypt; 3 Medical Molecular Genetics Department, Human Genetics and Genome Research Institute, National Research Centre, Cairo, Egypt

**Keywords:** Sanfilippo syndrome type A, *SGSH* gene, Heparan-N-sulphatase, Sulphamidase, MPS IIIA, Mucopolysaccharidosis IIIA

## Abstract

**Background:**

Lysosomal storage diseases (LSDs) is a large group of genetically heterogeneous inherited metabolic disorders that affect the functions of the lysosomes in various human tissues. Mucopolysaccharidosis type IIIA (MPSIIIA), Sanflippo syndrome A, is a rare autosomal recessive LSD caused by biallelic variants in the *SGSH* gene, codes for the lysosomal enzyme heparan-N-sulphatase. This study aimed to find out the SGSH mutational spectrum, clinical and biochemical characteristics in a cohort of MPS IIIA Egyptian patients.

**Results:**

Ten patients derived from 9 unrelated families, clinically and biochemically diagnosed having MPS IIIA secondary to heparan sulphatase deficiency, were enrolled. Patients, variably, displayed early-onset and progressive neurological and mental deterioration, aggressive and hyperactive behaviors, sleep disturbances and visceromegaly. Sanger sequencing of the *SGSH* coding and exon-intron boundaries revealed four homozygous disease-causing variants in all the patients (100%), three previously reported (p.Y224*, p.R377C, and p.V361Sfs*52), and a novel one (c.948delA; p.D317Tfs*96). The p.Y224* in exon 6 was the most recurrent variant (5/10, 50%), followed by the missense R377C in exon 8 (3/10; 30%), while the two frameshift truncating variants, each appeared in only one patient; presenting 10% of the disease causing variants.

**Conclusions:**

The pattern of variants recurrence in unrelated Egyptian patients highlights exons 6 and 8 as hot spots for first variant screening. The molecular findings of this study expand the *SGSH v*ariant spectrum and underline specific exons for first screening of MPS IIIA patients, which would largely help the early diagnosis and genetic counselling. To the best of our knowledge, the present study is the first delineating the *SGSH* variant profile in Egyptian Sanflippo A patients.

## Introduction

Sanfilippo syndromes or mucopolysaccharidosis type III (MPS III) is of the lysosomal storage diseases (LSDs) in which the basic metabolic pathology is the accumulation of the glycosaminoglycan heparan sulphate in lysosomes. There are four subtypes of Sanfilippo syndromes, MPS IIIA, MPS IIIB, MPS IIIC, and MPS IIID, Sanfilippo subtypes A, B, C, and D are associated with the deficiency of four different lysosomal enzymes that function in the degradative pathway of heparan sulphate: heparan-N-sulphatase (EC 3.10.1.1), α-Nacetylglucosaminidase (EC 3.2.1.50), acetyl-CoA N-acetyl transferase (EC 2.3.1.3), and N-acetylglucosamine-6- sulphatase (EC 3.1.6.14), respectively [1, 2].

The clinical manifestations of Sanfilippo A syndrome are mainly neurological, usually, occur after two years of apparently normal development and include hyperactivity, aggressive behaviour, developmental delay (particularly in speech), sleep disturbances, and hearing loss, in addition to the general features of MPS of coarse facies, hepatomegaly, hirsutism, diarrea, and skeletal involvements. The initial presentation is followed by a phase of progressive mental retardation and shortened life span; death usually occurs between the second and third decade of life [3]. Some rare cases of Sanfilippo A showed a slower progression and milder symptoms, resulting in a late diagnosis [4].

Sanfilippo A syndrome (MPS IIIA) is an autosomal recessive rare disease, caused by mutations in the N-sulfoglucosamine sulfohydrolase (*SGSH)* gene that lead to a deficiency of the heparan-N-sulphatase (sulphamidase) enzyme and subsequent accumulation of heparan sulphate (HS) and partially degraded products in the lysosomes of different organs. The accumulation of HS, misfolded proteins, monosialoganglioside (GM2), inflammatory cytokines interleukin-6 (IL-6) and impaired autophagy, in the brain, particularly the cerebral cortex are affecting several aspects of the neural processes, neural cell proliferation, neural axon guidance and synapse formation [5, 6].

The *SGSH* gene is located on chromosome 17q25.3, contains eight exons spanning approximately 11 Kb and encodes a protein of 502 amino acids. Sulfamidase protein that contains five potential N-glycosylation sites [7, 8].

The present study aims to identify the *SGSH* mutational spectrum in a cohort of Egyptian children patients with Sanfilippo Syndrome type A, who were ascertained based on the results of the biochemical enzyme deficiency.

## Subjects and methods

### Patients

**Fig. 1:**
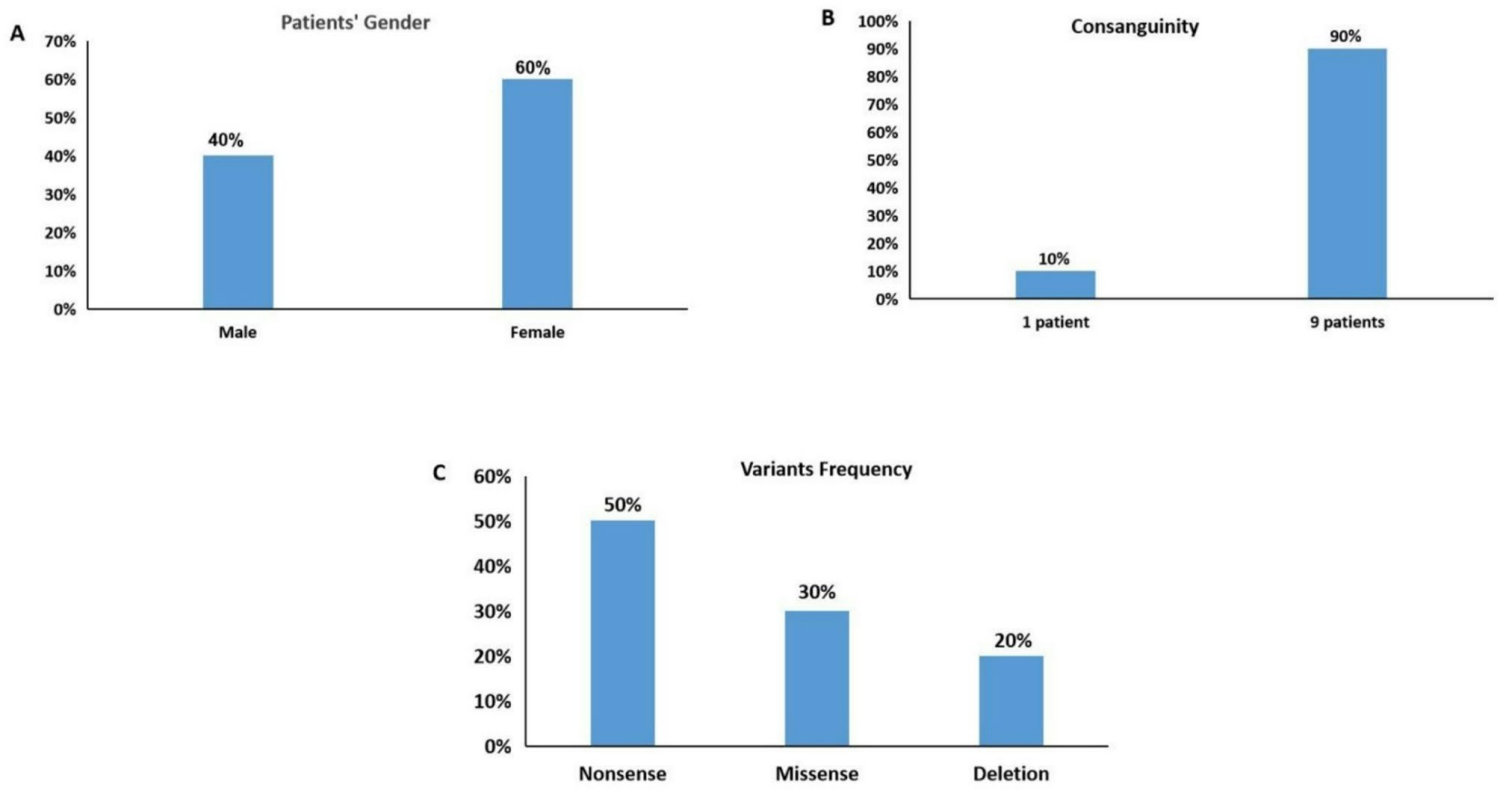
Patient Gender, Parental Consanguinity, and Variants Frequency in the MPS IIIA Study Group. **A**) Four males (40%) and six females (60%) patients, **B**) High consanguinity rate 90% (9/10) in Egyptian Sanfilippo type A patients, **C**) 50% (5/10) nonsense variants, 30% (3/10) missense variant, and 20% (2/10) deletion variant

Peripheral blood samples were obtained from 10 Sanfilippo syndrome type A Egyptian patients, (8 unrelated and two siblings), identified based on the specific enzyme deficiency. The study group involved 4 males and 6 females patients (Fig. 1A), their ages ranged from 2 to 9 years old. A positive family history was recorded in four patients. Parental consanguinity was found in 9 patients’ families (Fig. 1B). Written informed consent was obtained from the parents of the patients according to the guidelines of the Medical Research Ethics Committee of the National Research Centre (NRC) (approval no. 044101223).

### Biochemical analysis

Diagnosis of the MPS IIIA patients was basically done by the quantitative urinary glycosaminoglycans (GAGs) and qualitative (electrophoretic separation of GAGs) measurements [9]. High GAGs levels, for the age, were confirmed. The heparan & heparan sulfate (H&HS) spots were detected in the electrophoretic separation of GAGs. N-sulphoglucosamine sulphohydrolase enzyme activity determination was assayed in plasma using fluorogenic substrates [10].

### Molecular analysis

#### DNA isolation

Genomic DNA was extracted from the patients’ peripheral blood using the standard method [11]. The open reading frame and exon-intron boundaries of the *SGSH* gene were amplified using 9 overlapping primers, previously described [12]. Polymerase chain reaction (PCR) was done using 100 ng of genomic DNA, 25 pmol of each primer pair, 0.2 mmol/l dNTPs, 1.0 mmol/l MgCl, and 2.5 units DNA polymerase (Fermentas, EU- Thermo Scientific), and 1x NH4 reaction buffer (Thermo Scientific) [12]. Amplification conditions were as, initial denaturation 96 °C for 5 min, followed by 35 cycles of 96 °C for 1 min, annealing 60 –64 °C for 1 min, and 72 °C for 1 min and final extension 72 °C for 10 min. PCR products were purified using the QIAquick PCR purification kit (Qiagen, Hilden, Germany) according to the manufacturer’s protocol and bidirectional Sanger sequencing was run using the Big Dye Termination kit (Applied Biosystems, Foster City, California, USA). Variants were evaluated according to ACMG guidelines using NM_000199.5 as the reference transcript [13].

### Database and in-silico analyses

The identified disease’s relevant genetic variants were queried by browsing through different databases including the LOVD (Leiden Open Variation Database) [14], Human Gene Mutation Database (HGMD) (“HGMD^®^ home page,” 2023) [15] and NCBI dbSNP (database of Single Nucleotide Polymorphisms, ClinVar) [16]. Frequencies of the identified variants were checked against the 1000 genomes (“IGSR | samples,” 2023) [17], the gnomAD (“GLA | gnomAD,” 2023) databases [18], and Varsome database [19].

## Results

### Clinical findings

All patients presented the severe neurological phenotype of Sanfilippo syndrome type A. The patients were diagnosed between the age 2 and 9 years old. Parental consanguinity was present in 9 families of our study group. Patients’ clinical phenotype showed the cognitive impairment (10/10), hyperactivity (8/10), dysmorphic features (8/10), hirsutism (9/10), hepatospleenomegaly (5/10), skeletal abnormalities (5/10), deafness (5/10), and sleep disturbance (10/10). Patients presented progressive central nervous system (CNS) deterioration leading to the development of dementia. Developmental and speech delay, behavioral abnormalities, coarse faces, frequent upper-respiratory & ear infections, inguinal & umbilical hernias were of the clinical phenotype presented in our patients at variable degree of severity. Clinical details are shown in Table 1.

**Table 1:** The demographic, biochemical, and clinical characteristics of the affected patients with mucopolysaccaridosis type IIIA (MPS IIIA) in this study

Probands Characteristic	Patient1	Patient2	Patient3	Patient4	Patient5	Patient6	Patient7	Patient8	Patient9	Patient10
Gender	M	M	F	F	F	M	F	F	F	M
Age at diagnosis	**7yrs**	**5 (8/12)yrs**	**5yrs**	**5 (9/12)yrs**	**4 (8/12)yrs**	**9 (4/12)yrs**	**4yrs**	**1 (8/12)yrs**	**5 (6/12)yrs**	**6 (4/12)yrs**
Consanguinity	**+**	**-**	**+**	**+**	**+**	**+**	**+**	**+**	**+**	**+**
Family History	**-**	**-**	**-**	**+ sister**	**-**	**+sister**	**+sister**	**+ cousin**	**-**	**-**
**Biochemical Data**										
(H &HS) spots	**+**	**+**	**+**	**+**	**+**	**+**	**+**	**+**	**+**	**+**
GAGs (mg/mmol creatinine)	**14.2**	**5.7**	**20.5**	**23**	**18**	**34.9**	**21.4**	**58.3**	**22**	**10.8**
The reference range of SGSH activity (4-42.6 nmol/mg prot/hr)	**0.2**	**0.2**	**0.62**	**1.0**	**0.43**	**0.05**	**0.05**	**0.1**	**0.23**	**0.05**
**Clinical features**										
Hyperactivity	**+**	**+**	**+**	**+**	**+**	**--**	**+**	**--**	**+**	**+**
Speech delay	**+**	**+**	**+**	**+**	**+**	**+**	**+**	**+**	**+**	**+**
Developmental delay	**+**	**+**	**+**	**+**	**+**	**+**	**+**	**+**	**+**	**+**
Coarse fascies	**+**	**+**	**+**	**+**	**+**	**+**	**--**	**+**	**+**	**+**
Hepatospleenomegaly	**+**	**+**	**--**	**--**	**+**	**+**	**+** **spleenomegalay**	**+**	**--**	**--**
Joint stiffness	**+**	**+**	**--**	**Delayed milestones**	**+**	**+**	**--**	**Delayed milestones**	**Delayed** **walking**	**+**
Hearing loss	**Otitis media**	**Low lt ear hearing**	**--**	**--**	**--**	**Repeated otitis media**		**Ear shunt**	**Otitis media but good hearing**	**Low hearing & wear hearing device**
Teeth	**+** **Dental caries**	**--**	**+**	**--**	**--**	**+**	**+**	**+**	**+**	**+**
Hirshotism	**+**	**+**	**+**	**--**	**+**	**--**	**+**	**--**	**--**	**+**
Cornea	**--**	**--**	**+**	**--**	**--**	**+**	**--**	**--**	**--**	**--**

### Biochemical findings

The biochemical results revealed low heparan-N-sulphatase activity ranged between (0.05-1.0) nmol/mg prot/hr. All the patients showed high levels of urinary GAGs, ranging between 5.7 and 58.3 mg/mmol creatinine. Electrophoresis displays spots of Heparan and Heparan sulfate (H & HS). The concentration of N-sulphoglucosamine sulphohydrolase activity, GAGs, and electrophoretic pattern is shown in Table 1.

### Variant screening

**Fig. 2:**
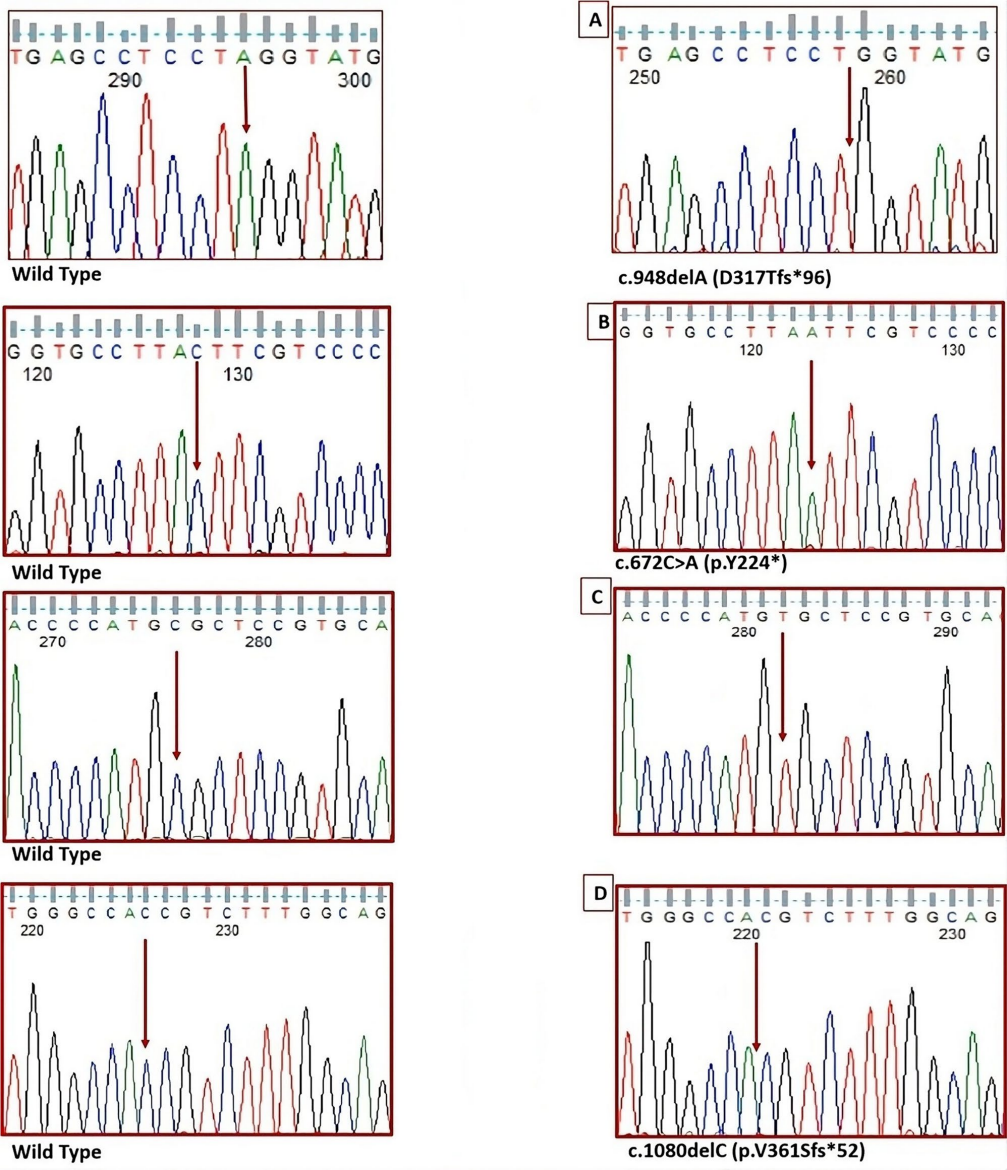
The sequencing electrophoregrams show a novel and three previously reported homozygous variants in the *SGSH* gene in Sanfilippo A syndrome patients in Egypt. (**A**) c.948delA (p.D317Tfs*96), (**B**) c.672 C > A (p. Y224*), (**C**) c.1129 C > T (p.R377C), and (**D**) c.1080delC (p.V361Sfs*52). The arrow indicates the site of variant (base changes and deletion)

Sanger sequencing was applied to amplify the eight exons and the intron-exons boundaries of the *SGSH* gene in nine overlapping fragments. The present study has identified a novel homozygous frameshift variant c.948delA (p.D317Tfs*96), and three previously reported homozygous variants; a nonsense, missense, and frameshift, c.672 C > A (p.Y224*), c.1129 C > T (p.R377C), and c.1080delC (p.V361Sfs*52) (Fig. 2). The variant details are shown in Table 2.

**Table 2:** The genotypes of variants in the *SGSH* gene in mucopolysaccharidosis type IIIA patients in this study

Patient ID	Exon no.	Alleles no.	Percentage	Variant	Nucleotide change	Type of Variant	Protein change	Mutation effect on protein	Ref.
**1**	6	2	50%	Y224*	c.672 C > A	Nonsense	Tyrosine to stop codon	Premature termination after 224 aa	[20]
**2**	6	2	50%	Y224*	c.672 C > A	Nonsense	Tyrosine to stop codon	Premature termination after 224 aa	[20]
**3**	6	2	50%	Y224*	c.672 C > A	Nonsense	Tyrosine to stop codon	Premature termination after 224aa	[20]
**4**	6	2	50%	Y224*	c.672 C > A	Nonsense	Aspartic acid to Thereonine	Premature termination after 224aa	[20]
**5**	7	2	10%	D317Tfs*96	**c.948delA** 1 bb deletion	Frameshift Deletion	Arginine to cysteine	Frameshift of premature termination at position 317 after 96 aa	**Novel**
**6**	8a	2	30%	R377C	c.1129 C > T	Missense	Arginine to cysteine	Non-conservative aa exchange	(37–39)
**7**	8a	2	30%	R377C	c.1129 C > T	Missense	Valine to Serine	Non-conservative aa exchange	(37–39)
**8**	8a	2	10%	V361Sfs*52	c.1080delC 1 bb deletion	Frameshift Deletion	Tyrosine to stop codon	Frameshift of premature termination at position 361 after 52 aa	(21,40)
**9**	6	2	50%	Y224*	c.672 C > A	Nonsense	Arginine to cysteine	Premature termination after 224aa	[20]
**10**	8a	2	30%	R377C	c.1129 C > T	Missense	Valine to Serine	Non-conservative aa exchange	(37–39)

#### The frameshift deletion variant

In this study, two homozygous frameshift deletion variants were identified. A novel one (c.948delA; p.D317Tfs*96), identified in exon 7 of the *SGSH* gene in only one patient number 5. This single base pair deletion (delA) at codon 317 resulting in the replacement of aspartic acid by threonine, which subsequently leads to a frameshift, premature termination of the encoded protein and a stop codon at 96 amino acids residues downstream. The c.948delA deletion frameshift (M_000199.5) was classified in Varsome as likely pathogenic [19].

This variant was not previously reported in the Human Gene Mutation Database (HGMD) or the Leiden Open Variation Database (LOVD).

The previously reported frameshift deletion variant (c.1080delC; p.V361Sfs*52); was in exon 8 of the *SGSH* gene and detected in one patient of the cohort number 8. This single base pair deletion (delC) replaces valine by serine at codon 361, which subsequently leads to a frameshift, premature termination and a stop codon 52 residues downstream (Table 2).

The c.1080delC (p.V361Sfs*52) deletion frameshift (M_000199.5) was reported in the publicly available mutational databases (Varsome.org) (rs770947426) [14–16, 18, 19] as a pathogenic variant on Chr17 at position 80,210,881 with a pathogenicity score of 17 and a conservation score of 7.992. This variant was not detected in exomes, gnomAD, or 1000 genome databases [17].

#### Nonsense variant

The previously reported nonsense variant (p.Y224*), detected in exon 6 of the *SGSH* gene in which tyrosine is substituted by a premature stop codon at codon 224, leading to the production of a truncated protein (Table 2) [20]. The p.Y224* was detected in five of the 10 patients numbers, 1, 2, 3, 4, and 10.

The nonsense c.672 C > A variant (M_000199.5), was classified as likely pathogenic with a pathogenicity score of 13 and a conservation score of 0.733 (Varsome.org) [14–16, 18, 19]. This variant was not detected in exomes, gnomAD, or 1000 genome databases [17].

#### Missense variant

The previously reported missense variant, p.R377C, was detected in exon 8 of the *SGSH* gene, in three of our patients, two sisters and another patient numbers, 6, 7, and 10 (Table 2).

The missense, c.1129 C > T, (p.R377C) (M_000199.5) variant located on Chr17 at position 80,210,832 was classified as a pathogenic variant, with a pathogenicity score of 19 and a conservation score of 5.346, Varsome (rs772311757) [14–16, 18, 19]. This variant was not detected in the exomes, gnomAD, or 1000 genome databases [17].

## Discussion

Mucopolysaccharidosis IIIA (MPS IIIA) known as Sanfilippo A syndrome is a lysosomal storage disorder (LSD) caused by the deficiency of the enzyme heparan sulfamidase (EC 3.10.1.1), which is necessary for the breakdown of the mucopolysaccharide heparan sulfate. MPS IIIA has the most severe and rapidly progressing disease course of the four MPS III subtypes [21, 22], while MPS IIIB is reported as the most common MPS III subtype among the diagnosed MPS III Egyptian patients [22].

Here, we report on the clinical, biochemical, and molecular characteristics of ten MPS IIIA patients from nine unrelated families. We identified four different mutations, a novel and three previously reported ones. Patients included in the present study follow the common severe phenotype observed in Sanfilippo A syndrome [1]. The clinical phenotype is predominated by progressive neurological degeneration with mental deterioration and behavioral abnormalities.

According to Nijmeijer study, MPS III symptoms typically start to show up at ages between 2 and 6 years [3]. The severely affected patients present their symptoms in the first year of life. In the mild-disease course, the symptoms begin in childhood or adolescence and are frequently under diagnosed, only when signs and symptoms become notable. Some rare cases had even slower progression and milder symptoms, which led to a delayed diagnosis [4].

In the present study, the patients’ age distribution revealed two age groups: age group I, with the manifestations started at 1–5 years old, four patients were allocated to this age group of early manifestation (40%) of MPS IIIA cases enrolled in this study. The age group II, the clinical presentations started at 5–10 years old, 6 of the 10 patients (60%) were in this second age group. The mean age at diagnosis was 5 years which is considered a late age of diagnosis of such serious disease with its progressive neurological damages (Table 1). Our findings with regard the first diagnostic age group were consistent with those of a previous Egyptian biochemical study, revealed that 65.7% of MPS IIIA patients had the mean diagnostic age at 5 years old and 9.6% of their cohort had an early diagnosis between the age of 3 to 12 month as they were already brothers and sisters of affected patients. Late diagnosis was reported in eighteen patients that were diagnosed at an age range of 6 to 17 years old; this late diagnosis may be due to a mild presentation of Sanfilippo syndrome or being the first diagnosed cases in the family [22].

Our patients’ sample showed a higher female to male ration (1.5:1), however, the small sample size may falsely give this ratio since in several other studies the male ratio was higher [23–29] (Table 1).

The high prevalence of consanguineous marriages in Egypt raises the risk of autosomal recessive disorders. In the present study, the consanguinity rate was as 90% among the parents of the MPS IIIA patients (Table 1). Genetic counselling that has been made possible with the present molecular genetic research is highly recommended for these families for any future pregnancies. In previous Egyptian studies, the consanguinity rates among MPS Egyptian patients ranged from 29.5 to 80% [23, 30]. Additionally, several previous Egyptian studies revealed a high consanguinity rate within MPS III patients [22, 24, 29, 31].

The central nervous system (CNS) damage was affirmed as the main presentation in our Sanfilippo patients. All patients seemed to have normal neurological functions, apart from the developmental delay, until the disease’s onset at 2 to 3 years old. But eventually all MPS IIIA patients had deteriorated CNS functions that progressively increased with age secondary to the accumulation of H/HS.

In the present study, 8 (80%) MPS IIIA patients suffered from aggressive behaviour and hyperactivity noted at the age of 3 years. The other two (20%) patients had different courses, one of them was too young (20 months) to develop the full signs of MPS IIIA syndrome, and the other one was at the final stage of the disease (bedridden).

The difficulty of audiometric data collection from MPS III patients due to their behavioral problems leads to inadequate recording of auditory function in MPS III syndrome [20]. In the present study, 5 (50%) MPS IIIA patients had general hearing problems and recurrent otitis media. The findings are consistent with others, reporting that 33.3% of MPS IIIA patients, 30.6% of MPS IIIB patients, and 13.6% of MPS IIIC or D patients had general ear problems [22].

All 10 patients (100%) displayed high urinary GAGs levels detected by Two-Dimensional Electrophoretic (2DE) abnormalities, the range read between (5.7–58.3) mg/mmol creatinine with the mean of 21 mg/mmol creatinine (Table 1). Electrophoritic patterns of Heparan and heparan sulfate (H & HS) abnormalities were detected in all (100%) MPS IIIA patients (Table 1). In a previous biochemical study, 242 out of 535 patients had high urinary GAGs and 233 were diagnosed as MPS patients because of abnormal 2DE; nine patients (3.7%) exhibited high GAG level, despite having normal 2DE, which is a common observation in many of such diseases [22, 29, 32]. In Fateen study, the mean urinary GAG level for 43 MPS III patients was 34.4 mg/mmol creatinine. The MPS III urinary GAG findings were classified into three groups based on patients’ age, 1–12 months, 1–5 years, and > 5 years, with the average of 48.8, 47.4, and 27 mg/mmol creatinine, respectively [33]. Additionally, the mean of urinary GAGs level in MPS IIIA patients was (25.6 mg/mmol creatinine), which was lower than that of MPS IIIB patients (47.5 mg/mmol creatinine). Seventy-three (31.3%) patients out of 233 diagnosed as MPS patients were diagnosed as MPS III because of the presence of (H & HS) spots in 2DE. This is considered a quite high percentage when compared to other studies. MPS III formed 17.3% of all MPS cases in Fateen (b) et al., and 28% in Fateen et al. [29, 33].

In the present study, all MPS IIIA patients had heparan-N-sulphatase (sulphamidase) (SGSH) enzyme deficiency and the range of enzyme activities in the affected patients was (0.2 to 1.0 nmol/mg prot/hr) with mean range of 0.29 ± 0.2 nmol/mg prot/17 h (Table 1). N-sulphoglucosamine sulphohydrolase enzyme activity results were slightly higher than those of the previous Egyptian study [22].

All the patients (100%) enrolled in this study were shown to carry homozygous variants in *SGSH*, five (50%) nonsense, three (30%) missense, and two (20%) frameshift deletion variants. The most common variant was p.Y244* (50%), identified in five MPS IIIA patients; the second most common variant was p.R337C (30%), identified in two sisters and another patient. Both truncating, frameshift deletions, the novel p.D317Tfs*96 and reported p.V361Sfs*52 occurred at low frequencies (10%) in our study group. The detected variants were distributed unequally along the exons of the *SGSH*. In 50% of cases (5/10), the disease-causing variant allocated in exons 6, 40% (4/10) in exon 8, and only one patient (10%) the causative mutation was in exon 7 (Fig. 1C). These findings highlighted exon 6 followed by exon 8 as potential mutational hotspots in Egyptian Sanfilippo A patients. This finding is consistent with a previous study for diagnosing MPS IIIA in the Korean population, which revealed that 50% of the diagnostic variants were located in exon 6, and the other 50% were detected in exon 8 of the *SGSH* [34].

### Truncating frameshift variants

#### The novel mutation

In our study, a novel single base deletion variant (NM_000199.5):c.948delA, (p.D317Tfs*96) resulting in frameshift and premature termination of the encoded protein was identified in only one patient of the study group. It is anticipated that this mutation will produce a non-functioning truncated protein. This variant was associated with attenuated clinical symptoms and the patient was diagnosed at the age of 4 years old. The other single base deletion variant, c.1080delC (p.V361Sfs*52), detected in one of our MPS IIIA patient was previously reported for the first time, in a Tunisian patient [35], and by others [25].

#### Nonsense variant

The nonsense variant, (NM_000199.5):c.672 C > A (p.Y224*), was the most common variant identified in Egyptian MPS IIIA patients of the present study. This variant was previously reported only once in a homozygous status in a patient presented a severe phenotype as defined by the residual enzyme activity, at the National Referral Laboratory at the Women’s and Children’s Hospital, SA, Australia [20]. The study investigators had reprogrammed the patients` skin fibroblasts into induced pluripotent stem cells (iPSCs). The generation of MPS IIIA iPSCs and neural progenitor cells (NPCs) provides an early insight into the mechanism of CNS dysfunction in MPS IIIA. The reduction in HS GAG turnover and associated disruption to lysosomal function and accumulation of unique HS GAG structures in MPS IIIA do not impressively affect iPSC generation from MPS IIIA skin fibroblasts or their differentiation to NPCs. However, MPS IIIA iPSC derived NPC proliferation is affected by GAG accumulation that obstructs FGF2 signaling. Furthermore, the formation of neurons from MPS IIIA iPSC-derived NPCs and/or their survival was reduced via an as yet unidentified mechanism [20].

#### Missense variants

The second common variant, c.1129 C > T (p.R377C) was identified in three patients numbers, 6, 7, and 10 of the MPS IIIA patients’ group in a homozygous form. This missense variant leads to substitution of arginine residue, a basic, polar, and less hydrophobic amino acid that serves as the precursor of the free radical nitric oxide (NO), by cysteine residue, an acidic and polar amino acid. The missense R377C variant was previously reported in a Tunisian patient in heterozygous form [35].

The missense R377C variant affects the highly mutable CpG dinucleotides, and the replacement of nucleotide C to T is consistent with “methylation-mediated deamination” of 5-methylcytosine as one of the potential mutagenesis pathways. Several arginine residues seem to be “hot spots” for these variants. In a previous study, missense R377H and R377L variants at the same codon but a different residue substitution suggested that the residue R377 is highly conserved.

#### Recurrent variant

The most common variant identified in the MPS IIIA patients was p.Y224* (5/10), which detected at a high frequency of 50% of the mutant alleles in the Egyptian MPS IIIA patients. The second most common variant was p.R337C (30%), identified in two sisters and another patient of the study group.

Identifying a single recurrent variant in a specific population may indicate a founder effect, which greatly aids in the early diagnosis and carrier detection of Sanfilippo A syndrome in the Egyptian population. Early diagnosis plays a significant role in genetic counseling of affected families and prenatal diagnosis in future pregnancies.

### Genotype-phenotype correlation

A genotype-phenotype correlation was observed in our cohort of mucopolysaccharidosis type IIIA (MPS IIIA) patients. This finding suggests a potential direct relationship between the molecular genetic defect and the clinical symptoms they experienced. However, variability in clinical presentation in patients who carry the same gene mutation highlights the role of modifying factors in MPS IIIA patients. The genotypic-phenotypic correlation is crucial for predicting disease severity and course progression in these patients.

The p.Y224* nonsense variant was identified in five patients (P1, P2, P3, P4, and P9), all of whom presented with the common clinical features of MPS IIIA, including hyperactivity, coarse facial features, and speech and developmental delay. However, the other associated clinical features revealed notable variability; the hepatosplenomegaly (HSM) and severe joint stiffness were reported in P1 and P2. In contrast, patients P4 and P9 primarily exhibited delayed milestones without severe joint contractions. Recurrent otitis media was documented in patients P1, P2, and P9, causing defective hearing in patient P2. Dental caries were present in patients P1, P3, and P9. Corneal affection was an isolated finding in patient P3. Hirsutism was noted in patients P1, P2, and P3. Interestingly, patient P2 demonstrated the most severe progression and is currently presenting as bedridden with recurrent suffocation secondary to significant sputum production and chest infections.

The R377C missense variant was identified in three patients (P6, P7, and P10), all of whom presented with dental decay, speech delay, and global developmental delay. Other clinical features revealed phenotypic variability among these patients; hyperactivity and hirsutism were observed in patients P7 and P10. Coarse facial features and joint stiffness were noted in patients P6 and P10. Regarding organomegaly, P6 exhibited hepatosplenomegaly (HSM), while P7 presented with splenomegaly alone. Recurrent otitis media was a common complication in patients P6 and P10, with patient P10 additionally suffering from hearing impairment. The clinical course of patient P6 was particularly severe, characterized by recurrent seizures (epilepsy), bedridden, and reliance on enteral nutrition. The family of patient P6 expressed significant apprehension regarding her prognosis, including concerns about respiratory compromise, and reported similar anxieties regarding the anticipated progression of her younger, affected sibling.

Patient P8 was identified with the V361Sfs*52 frameshift deletion variant. Uncharacteristically for Mucopolysaccharidosis type IIIA (MPS IIIA) patients, patient P8 did not exhibit hyperactivity, hirsutism, or corneal cloudiness. However, consistent with MPS IIIA, presented with speech and developmental delay, coarse facial features, and hepatosplenomegaly (HSM). Additional manifestations included delayed milestones, dental decay, and recurrent otitis media, for which an ear shunt had been placed.

Patient P5 was identified with the novel frameshift deletion variant, D317Tfs*96. This patient presented with a comprehensive set of common clinical manifestations characteristic of mucopolysaccharidosis type IIIA (MPS IIIA) syndrome. These included hyperactivity, coarse facial features, hepatosplenomegaly (HSM), hirsutism, and speech and developmental delay. Notably, despite the widespread clinical involvement, P5 did not exhibit impairment in hearing, corneal affection, or dental anomalies.

## Conclusions

The current study presents the first genetic analysis of the *SGSH* gene in Egyptian patients with Sanfilippo A syndrome. Gene sequencing of the ten Egyptian patients has detected a novel homozygous deletion variant, D317Tfs*96, and three previously reported homozygous variants, Y224*, R377C, and V361Sfs*52. The most common variant, Y224*, was identified in half of the tested MPS IIIA patients group, and to our knowledge, this is the second time it has been detected around the world. A larger cohort of MPS IIIA Egyptian patients need to be tested to confirm that this variant is specifically common in the Egyptian population and could be used as a primary for variant testing. Study findings will help in the early-diagnosis of Sanfilippo A syndrome, carrier testing, and improve genetic counselling in the Egyptian population.

The analysis of genetic variants and their phenotypic effects reveals a spectrum of disease severity among patients. Both the p.Y224* nonsense and p.R377C missense variants are associated with the greatest phenotypic impact, seen in patients P2 and P6, respectively. These patients presented severe progression of the MPS IIIA phenotype, characterized by recurrent seizures (epilepsy), being bedridden with recurrent suffocation, and reliance on enteral nutrition. Additionally, the novel p.D317Tfs*96 frameshift deletion variant is associated with a comperhensive set of common clinical features typical of MPS IIIA in patient P5. Conversely, the p.V361Sfs*52 frameshift deletion variant corresponds to the least phenotypic effect, observed in patient P8, who shows an atypical MPS IIIA clinical presentation, indicating a milder form of the disease with this specific frameshift mutation. This variation emphasizes how different types of variants (nonsense, missense, and frameshift) can alter protein function and influence the manifestation of MPS IIIA in the Egyptian population.

## Data Availability

Sequencing and biochemical data generated in this study are available upon a reasonable request made to the corresponding authors.

## References

[1] Valstar MJ, Ruijter GJG, van Diggelen OP, Poorthuis BJ, Wijburg FA. Sanfilippo syndrome: a mini-review. J Inherit Metab Dis. 2008;31(2):240–52. 18392742 10.1007/s10545-008-0838-5

[2] Andrade F, Aldámiz-Echevarría L, Llarena M, Couce ML. Sanfilippo syndrome: overall review. Pediatr Int. 2015;57(3):331–8. 25851924 10.1111/ped.12636

[3] Nijmeijer SC, van den Born LI, Kievit AJ, Stepien KM, Langendonk J, Marchal JP, et al. The attenuated end of the phenotypic spectrum in MPS III: from late-onset stable cognitive impairment to a non-neuronopathic phenotype. Orphanet J Rare Dis. 2019;14:1–10. 31718697 10.1186/s13023-019-1232-0 PMC6852993

[4] Muschol N, Pohl S, Meyer A, Gal A, Ullrich K, Braulke T. Residual activity and proteasomal degradation of p. Ser298Pro sulfamidase identified in patients with a mild clinical phenotype of Sanfilippo A syndrome. Am J Med Genet A. 2011;155(7):1634–9. 10.1002/ajmg.a.34053 21671382

[5] Dwyer CA, Scudder SL, Lin Y, Dozier LE, Phan D, Allen NJ, et al. Neurodevelopmental changes in excitatory synaptic structure and function in the cerebral cortex of Sanfilippo syndrome IIIA mice. Sci Rep. 2017;7(1):46576. 28418018 10.1038/srep46576 PMC5394534

[6] Viana GM, Priestman DA, Platt FM, Khan S, Tomatsu S, Pshezhetsky AV. Brain pathology in mucopolysaccharidoses (MPS) patients with neurological forms. J Clin Med. 2020;9(2):396. 32024172 10.3390/jcm9020396 PMC7073982

[7] Scott HS, Blanch L, Guo XH, Freeman C, Orsborn A, Baker E, et al. Cloning of the sulphamidase gene and identification of mutations in Sanfilippo A syndrome. Nat Genet. 1995;11(4):465–7. 7493035 10.1038/ng1295-465

[8] Karageorgos LE, Guo XH, Blanch L, Weber B, Anson DS, Scott HS, et al. Structure and sequence of the human sulphamidase gene. DNA Res. 1996;3(4):269–71. 8946167 10.1093/dnares/3.4.269

[9] Whiteman P, Henderson H. A method for the determination of amniotic-fluid glycosaminoglycans and its application to the prenatal diagnosis of hurler and Sanfilippo diseases. Clin Chim Acta. 1977;79(1):99–105. 408055 10.1016/0009-8981(77)90466-1

[10] Marsh J, Fensom A. 4-methylumbelliferyl α‐N‐acetylglucosaminidase activity for diagnosis of Sanfilippo B disease. Clin Genet. 1985;27(3):258–62. 3921297 10.1111/j.1399-0004.1985.tb00217.x

[11] Miller SA, Dykes DD, Polesky HF. A simple salting out procedure for extracting DNA from human nucleated cells. Nucleic Acids Res. 1988;16(3):1215. 3344216 10.1093/nar/16.3.1215 PMC334765

[12] Beesley CE, Young EP, Vellodi A, Winchester BG. Mutational analysis of Sanfilippo syndrome type A (MPS IIIA): identification of 13 novel mutations. J Med Genet. 2000;37(9):704–7. 11182930 10.1136/jmg.37.9.704 PMC1734705

[13] Richards S, Aziz N, Bale S, Bick D, Das S, Gastier-Foster J, et al. Standards and guidelines for the interpretation of sequence variants: a joint consensus recommendation of the American college of medical genetics and genomics and the association for molecular pathology. Genet Med. 2015;17(5):405–23. 25741868 10.1038/gim.2015.30 PMC4544753

[14] Fokkema IF, Taschner PE, Schaafsma GC, Celli J, Laros JF, den Dunnen JT. LOVD v. 2.0: the next generation in gene variant databases. Hum Mutat. 2011;32(5):557–63. 21520333 10.1002/humu.21438

[15] Stenson PD, Ball EV, Mort M, Phillips AD, Shiel JA, Thomas NS, et al. Human gene mutation database (HGMD^®^): 2003 update. Hum Mutat. 2003;21(6):577–81. 12754702 10.1002/humu.10212

[16] Sherry ST, Ward MH, Kholodov M, Baker J, Phan L, Smigielski EM, et al. DbSNP: the NCBI database of genetic variation. Nucleic Acids Res. 2001;29(1):308–11. 11125122 10.1093/nar/29.1.308 PMC29783

[17] 1000 Genomes Project Consortium. A global reference for human genetic variation. Nature. 2015;526(7571):68. 26432245 10.1038/nature15393 PMC4750478

[18] Chen S, Francioli LC, Goodrich JK, Collins RL, Kanai M, Wang Q et al. A genome-wide mutational constraint map quantified from variation in 76,156 human genomes. BioRxiv. 2022;2022–03.

[19] Kopanos C, Tsiolkas V, Kouris A, Chapple CE, Aguilera MA, Meyer R, et al. VarSome: the human genomic variant search engine. Bioinformatics. 2019;35(11):1978. 30376034 10.1093/bioinformatics/bty897 PMC6546127

[20] Lehmann RJ, Jolly LA, Johnson BV, Lord MS, Kim HN, Saville JT, et al. Impaired neural differentiation of MPS IIIA patient induced pluripotent stem cell-derived neural progenitor cells. Mol Genet Metab Rep. 2021;29:100811. 34712574 10.1016/j.ymgmr.2021.100811 PMC8531667

[21] Valstar MJ, Neijs S, Bruggenwirth HT, Olmer R, Ruijter GJ, Wevers RA, et al. Mucopolysaccharidosis type IIIA: clinical spectrum and genotype-phenotype correlations. Ann Neurol. 2010;68(6):876–87. 21061399 10.1002/ana.22092

[22] Nosier SS, El Nakeeb SM, Ibrahim MM, El-Gammal M, Fateen EM. Biochemical diagnosis of Sanfilippo disorder types A and B. J Genet Eng Biotechnol. 2023;21(1):112. 37947910 10.1186/s43141-023-00586-7 PMC10638229

[23] Shawky RM, Zaki EA, Fateen EM, Refaat M, Eldin N. Profile of Egyptian patients with mucopolysaccharidosis. Egypt J Med Hum Genet. 2008;9(1):11–22.

[24] Fateen EM, Gouda AS, Ibrahim MM, Abdallah ZY. Fifteen years experience: Egyptian metabolic lab. Egypt J Med Hum Genet. 2014;15(4):379–85.

[25] Elmonem MA, Mahmoud IG, Mehaney DA, Sharaf SA, Hassan SA, Orabi A, et al. Lysosomal storage disorders in Egyptian children. Indian J Pediatr. 2016;83:805–13. 26830282 10.1007/s12098-015-2014-x

[26] Selim L, Abdelhamid N, Salama E, Elbadawy A, Gamaleldin I, Abdelmoneim M, et al. Cardiovascular abnormalities in Egyptian children with mucopolysaccharidoses. J Clin Diagn Res JCDR. 2016;10(11):SC05. 28050459 10.7860/JCDR/2016/21135.8851 PMC5198412

[27] Turkia B, Tebib N, Azzouz H, Abdelmoula MS, Chehida B, Chemli J, et al. Incidence of mucopolysaccharidoses in Tunisia. Tunis Med. 2009;87(11):782–5. 20209839

[28] AlObaidy H. Patterns of inborn errors of metabolism: A 12 year single-center hospital-based study in Libya. Qatar Med J. 2014;2013(2):18. 10.5339/qmj.2013.18 PMC4080488 25003067

[29] Fateen E, Abdallah ZY, Nazim WS, Ibrahim M, Radwan A. Mucopolysaccharidoses diagnosis in the era of enzyme replacement therapy in Egypt. Heliyon. 2021;7(8). 10.1016/j.heliyon.2021.e07830 PMC8387752 34471711

[30] Afifi H, El-Ruby M, El-Bassyouni H, Ismail S, Aglan M, El-Harouni A, et al. The most encountered groups of genetic disorders in Giza governorate, Egypt. Bratisl Lek Listy. 2010;111(2):62–9. 20429316

[31] Temtamy S, Aglan M. Consanguinity and genetic disorders in Egypt. Middle East J Med Genet. 2012;1(1):12–7.

[32] Tomatsu S, Okamura K, Maeda H, Taketani T, Castrillon S, Gutierrez M, et al. Keratan sulphate levels in mucopolysaccharidoses and mucolipidoses. J Inherit Metab Dis. 2005;28(2):187–202. 15877208 10.1007/s10545-005-5673-3

[33] Fateen EM, Ibrahim MM, Gouda AS, Youssef ZA. Biochemical diagnosis of mucopolysaccharidoses over 11 years: the Egyptian experience. Middle East J Med Genet. 2014;3(1):16–23.

[34] Kim MS, Yang A, Noh E, seon, Kim C, Bae GY, Lim HH, et al. Natural history and molecular characteristics of Korean patients with mucopolysaccharidosis type III. J Pers Med. 2022;12(5):665. 35629088 10.3390/jpm12050665 PMC9145712

[35] Ouesleti S, Brunel V, Turkia HB, Dranguet H, Miled A, Miladi N, et al. Molecular characterization of MPS IIIA, MPS IIIB and MPS IIIC in Tunisian patients. Clin Chim Acta. 2011;412(23–24):2326–31. 21910976 10.1016/j.cca.2011.08.032

